# The neural and genetic correlates of satisfying sexual activity in heterosexual pair‐bonds

**DOI:** 10.1002/brb3.1289

**Published:** 2019-05-14

**Authors:** Bianca P. Acevedo, Michael J. Poulin, Glenn Geher, Scott Grafton, Lucy L. Brown

**Affiliations:** ^1^ University of California at Santa Barbara Santa Barbara California; ^2^ State University of New York at Buffalo Buffalo New York; ^3^ State University of New York at New Paltz New Paltz New York; ^4^ Department of Neurology Albert Einstein College of Medicine Bronx New York

**Keywords:** fMRI, oxytocin, pair‐bonding, prefrontal cortex, sexual frequency, sexual satisfaction, vasopressin

## Abstract

**Introduction:**

In humans, satisfying sexual activity within a pair‐bond plays a significant role in relationship quality and maintenance, beyond reproduction. However, the neural and genetic correlates for this basic species‐supporting function, in response to a pair‐bonded partner, are unknown.

**Methods:**

We examined the neural correlates of oxytocin‐ (*Oxtr* rs53576) and vasopressin‐ (*Avpr*1a rs3) receptor genotypes with sexual satisfaction and frequency, among a group of individuals in pair‐bonds (M relationship length = 4.1 years). Participants were scanned twice (with functional MRI), about 1‐year apart, while viewing face images of their spouse and a familiar, neutral acquaintance.

**Results:**

Sex satisfaction scores showed significant interactions with *Oxtr* and *Avpr* variants associated with social behaviors in a broad network of regions involved in reward and motivation (ventral tegmental area, substantia nigra [SN], and caudate), social bonding (ventral pallidum), emotion and memory (amygdala/hippocampus), hormone control (hypothalamus); and somatosensory and self‐other processing (SII, frontal, and temporal lobe). Sexual frequency interactions also showed activations in the SN and paraventricular hypothalamus for *Avpr*, and the prefrontal cortex for *Oxtr*.

**Conclusions:**

Satisfying sexual activity in pair‐bonds is associated with activation of subcortical structures that support basic motivational and physiological processes; as well as cortical regions that mediate complex thinking, empathy, and self‐other processes highlighting the multifaceted role of sex in pair‐bonds. *Oxtr* and *Avpr* gene variants may further amplify both basic and complex neural processes for pair‐bond conservation and well‐being.

## 1 INTRODUCTION

In humans, sexuality clearly extends beyond reproductively relevant acts (Peterson, Geher, & Kaufman, 2011) to functions related to pair‐bonding and partner preference. For example, in addition to heterosexual vaginal intercourse, that takes place during ovulation, humans, unlike nearly all other mammals, often engage in sex outside of ovulation. Humans also engage in nongenital sexual activities, such as romantic kissing—which is thought to serve important mate‐assessment functions through the trading of salivary samples and pheromones. These nongenital sexual activities are thought to provide information about the potential partner's health, time of ovulatory cycle, and even commitment, as individuals who refuse to spend time kissing may be signaling that they are not good candidates for sexual intimacy and long‐term mate‐ships (Geher & Kaufman, 2014; Hughes, Harrison, & Gallup, 2007). Also, research suggests that female orgasm and the propensity for women to initiate sex are associated with a preference for males with high family incomes, sense‐of‐humor, intelligence, and determination—qualities that increase benefits for successful offspring (Gallup, Ampel, Wedberg, & Pogosjan, 2014).

Satisfying sex also seems to promote relationship longevity. For example, across five‐countries research showed that satisfying sex in marriages was associated with lower reports of extramarital affairs (Nowak & Danel, 2014). Sexual interest, activity, and satisfaction have also been shown to be positively associated with good health in middle‐age and late‐life (Matthais, Lubben, Atchison, & Schweitzer, 1997). On the other hand, sexual dissatisfaction or apathy toward sex may be a source or symptom of distress in marriage (Perel, 2007). In turn, marital dissatisfaction is associated with diminished well‐being, such as lowered immunity (Jaremka, Glaser, Malarkey, & Kiecolt‐Glaser, 2013).

Sexual frequency advantages have been harder to discern (Schoenfeld, Loving, Pope, Huston, & Štulhofer, 2017). For example, McNulty, Wenner, and Fisher (2016) examined 207 married couples over 4‐years and found that sexual frequency did not predict increases or changes in self‐reported marital satisfaction over time. Likewise, Loewenstein, Krishnamurti, Kopsic, and McDonald (2015) investigated 130 married couples over 3 months and found that doubling the frequency of sexual intercourse had no positive effect on self‐reported marital quality. However, studies, including those with large representative samples, have shown that there is a strong correlation between frequency of penile‐vaginal intercourse and relationship satisfaction (Brody, Costa, Klapilová, & Weiss, 2018). Thus, some researchers have proposed that evolutionary processes should favor the one sexual behavior (penile‐vaginal sex) that could potentially result in reproduction. Correspondingly, studies show that compared with masturbation‐induced orgasm, penile‐vaginal intercourse is associated with well‐being including reduced alexithymia, particularly in females (Brody, 2003); vagal tone indices associated with longevity, emotion regulation, and social bonding (Costa & Brody, 2012); and neuro‐hormonal benefits (Leeners et al., 2013). Moreover, sexual activity in couples has been shown to be positively associated with enhanced mood, stress‐reduction, and meaning in life (Brody, 2006; Kashdan, Disabato, & Short, 2017; Kashdan, Adams, Kashdan, & Riskind, 2013). Finally, research suggests that sex's health benefits may be mediated by releases in dopamine and oxytocin (Meston & Frohlich, 2000). Thus, it is important to investigate both implicit and explicit psychological and physiological measures of sex behaviors (Hicks, McNulty, Meltzer, & Olson, 2016).

Neuroimaging studies of sexual arousal have consistently shown activation of brain regions involved in reward, motivation, emotion, autonomic, neuroendocrine, and sensory processes (i.e., the SN, striatum, amygdala, parietal, temporal, and prefrontal regions; and the insula) (Poeppl et al., 2016; Stoléru, Fonteille, Cornélis, Joyal, & Moulier, 2012). As such, some researchers have proposed that sexual mate preference may be largely coordinated by phylogenetically old, subcortical brain structures that mediate reward, emotion, and attention; along with cortical regions that coordinate high‐order cognitive processes, such as self‐reflection and decision‐making (Stoléru et al., 2012). Somewhat similarly, human and animal studies have identified subcortical dopamine, oxytocin‐, opioid‐, and vasopressin‐rich regions as critical for pair‐bonding (Acevedo, Aron, Fisher, & Brown, 2012; Aron et al., 2005; Bartels & Zeki, 2000); as well as cortical areas for cognitive and self‐reflective processes in the context of human romantic love (Song et al., 2015).

Studies have also implicated the neuropeptides oxytocin (OT) and arginine‐vasopressin (AVP) in a variety of social behaviors including trust, cooperation, pair‐bonding, reproduction, maternal care, facial recognition, and the regulation of aggression toward strangers (Auyeung et al., 2015; Bartz, Zaki, Bolger, & Ochsner, 2011; Brunnlieb et al., 2016; Insel & Shapiro, 1992; Lee, Macbeth, Pagani, & Young, 2009; Meyer‐Lindenberg, 2008; Wang, Young, Vries, & Insel, 1999; Young, Lim, Gingrich, & Insel, 2001). These effects have also been shown to be associated with the oxytocin‐ (*Oxtr rs53576)* and vasopressin‐ receptor (*Avpr1a rs3)* genotypes. The *Oxtr rs53576* marker is a single‐nucleotide polymorphism (SNP) of the *Oxtr* gene that results in individuals having zero, one, or two G‐alleles (vs. A‐alleles). Studies have shown that individuals with a greater number of G (vs. A) alleles display greater empathy, altruism, sensitive parenting, and sociality (Buffone & Poulin, 2014; Li et al., 2015; Poulin, Holman, & Buffone, 2012; Rodrigues, Saslow, Garcia, John, & Keltner, 2009; Uzefovsky et al., 2015). It is not known, however, if allele variability reflects a greater number of OT receptors or greater sensitivity to OT.

Similarly, the *Avpr1a rs3* variant consists of length variation in a repetitive stretch of the *Avpr1a* gene. The longer alleles were found to correlate with more stable pair‐bonds, greater altruism, age of first intercourse, amygdala activation during an emotional face recognition task, and hippocampal volume (Brunnlieb et al., 2016; Knafo et al., 2008; Meyer‐Lindenberg, 2008; Poulin et al., 2012; Walum et al., 2008). Also, there is some evidence that longer alleles correspond to greater density of *Avpr1a* receptors (Knafo et al., 2008). However, studies have not examined their relation to sexual variables in coupled individuals which would provide evidence of endogenous mechanisms for pair‐bond maintenance that may be phylogenetically conserved, and that have been rarely described in humans.

Still, questions remain with respect to the neural mechanisms underlying sexual arousal in humans. For example, hypothalamic activation has been shown for males, but not females; and for younger males not older males (Hamann, Herman, Nolan, & Wallen, 2004; Stoleru, Ennaji, Cournot, & Spira, 1993). This may be due to the nature of stimuli used, as individuals differ with respect to the sorts of things that they find sexually arousing (Rupp & Wallen, 2008), including whether partner‐specific stimuli were tested. Also, studies of sexual arousal in humans have largely relied on general visual sexual stimuli, perhaps without awareness to data suggesting that pornography may actually undermine sexual satisfaction in couples (Yucel & Gassanov, 2010).

Thus, to understand the neural and hormonal genetic markers underlying sexual satisfaction and frequency within pair‐bonds, we imaged the brains (with functional MRI) of individuals in a first‐time marriage (twice, about 1‐year apart) in response to images of the spouse (vs. a familiar, neutral acquaintance). Participants were scanned twice to examine attachment‐related changes for another study, but in the present research, follow‐up scans allowed us to investigate replicated neural effects. We examined correlations of neural responsivity to images of the spouse (vs.theacquaintance) with self‐reports of sexual satisfaction and frequency, and their interactions with *Oxtr rs53576* and *Avpr1a rs3* genotype variants. Thus, this was the first study to examine the interaction of sexual satisfaction in marriages with genetic markers and neural activity in response to partner‐specific stimuli. Thus, we intended to highlight biological markers associated with this basic species evolutionary function for mating, parenting, and family stability throughout the lifespan (Donnelly, 1993; Geher & Kaufman, 2014; Nowak & Danel, 2014; Young & Wang, 2014). These markers may also provide insight on potential therapeutic targets for a variety of sexual, relationship, and addiction issues.

## 2 METHODS

### 2.1 Participants

This study was approved by the Human Subjects committees at the University of California, Santa Barbara (UCSB) and Albert Einstein College of Medicine. Participants were recruited by newspapers, internet ads, and flyers seeking, “newlywed and engaged couples.” Eligibility criteria were: marriage to a first‐time spouse, no children, relationship length (<7 years), overall good health, no fMRI contraindications, nonuse of anti‐depressants or excessive medications, and no major surgeries. All participants provided informed consent and received payment.

Participants completed fMRIs and surveys at two visits. 1‐year apart. At Time 1 (T1) partcipants were 18 (10 women) healthy, right‐handed individuals, ages 21 to 32 (*M* = 27.2, *SD* = 3.4), in committed relationships (*M* = 4.1 years, *SD* = 2.8), soon‐to‐be or recently married with a mean annual household income of $60,000 (range $16,000 to $110,000). Two participants had earned a high‐school degree, 10 a college‐degree, and 6 a M.A. or higher. The ethnic composition of the sample was as follows: three Asian‐American, three Hispanic/Latino, and 12 White/European‐American. At Time 2 (T2), 13 (seven women) subjects completed fMRIs (M age = 28.4 years, *SD* = 3.4; relationship lengths *M* = 5.9 years, *SD* = 2.9).

### 2.2 Procedure

Before scanning, all participants were interviewed to determine an appropriate *highly‐familiar neutral acquaintance (HFN)* **,** matched to the Partner by gender, age, and length of time known. The HFN served as a control for facial familiarity for the imaging analyses. Subjects were asked to provide facial photos of the Partner and HFN which were digitized according to standard procedures, and were displayed using Presentation software (Psychological Software Tools, Inc.).

#### 2.2.1 fMRI protocol

The fMRI protocol consisted of a 12‐min session where participants viewed alternating Partner and HFN images (displayed for 20‐s each; six repetitions). Participants were instructed to think about the Partner or HFN (*not* sexually) while viewing each face image. To reduce carry‐over effects of viewing the face images, stimuli were followed by a countback task, where subjects were asked to mentally count backwards in increments of seven, starting with a random four‐digit number displayed on the screen. Identical photos were used at T1 and T2. Participants were debriefed and provided emotional ratings using a button‐box while still in the scanner, confirming appropriate emotion elicitation corresponding with the target stimulus (Acevedo et al., 2014).

#### 2.2.2 Genetic sampling

Subjects provided saliva samples for DNA extraction via Oragene test tubes (http://www.dnagenotek.com). Saliva was analyzed for the *Oxtr rs53576* and *Avpr1a* rs3 genotype variants. Genotyping of the *Oxtr* rs53576 single nucleotide polymorphism (SNP) was conducted with MassARRAY Compact system genotyping technology (Assays‐by‐Design) on a panel of custom SNP assays designed using RealSNP and MassARRAY Assay Designer (Sequenom Inc). The protocol involved PCR amplification of 10 ng DNA using SNP specific primers followed by a base extension reaction using iPLEX Gold chemistry (Sequenom Inc.). The final base extension products were treated and spotted on a 384‐pad SpectroCHIP using a ChipSpotter LT nanodispenser (Samsung). A MassARRAY Analyzer Compact MALDI‐TOF‐MS was used for the data acquisition process from the SpectroCHIP. The resulting genotypes were called using MassARRAY Typer Analyzer v4.0 (Sequenom Inc.), and the number of G alleles (0–2) was used as a continuous variable in our analyses. For the *Avpr1a* rs3 polymorphism the number of repeat sequences was identified via fragment analysis. In this technique, a repeat sequence is specified using a sequence‐specific primer congregated to a fluorescent probe and amplified for detection using polymerase chain reaction (PCR). Microsatellite fragment analysis of *Avpr1a* rs3 was conducted through capillary electrophoresis (Sequenom, Inc.). For *Avpr1a*, 6‐FAM and HEX labeled PCR products were mixed and analyzed in multiplex. Samples were denatured at 92°C for 2‐min and then cooled to 4°C on a MJ Research PT‐100 Peltier Thermal Cycler. Samples were transferred to a 96‐well sequencing plate and assigned well coordinates using Applied Biosystems Foundation Data Collection Version 3.0. Electrophoresis of the samples was performed with the Applied Biosystems 3130xl Prism Genetic Analyzer, using dye/filter set, DS‐30. The data were processed and analyzed using Applied Biosystems GeneMapper Software Version 3.7. Control samples were sequenced to determine the repeat‐size based on total fragment length. A left and right offset of 0.4 was used in the bin set parameter of the GeneMapper software to set the limits for acceptable fragment migration for each repeat. The number of repeat sequences was categorized as “long” versus “short” that were coded as the number of long alleles (0–2) as was done in prior research on pro‐social behavior (Knafo et al., 2008; Poulin et al., 2012).

#### 2.2.3 Questionnaires

Participants completed the following questionnaires: (a) the Relationship Assessment Scale (RAS; Hendrick, 1988), a seven‐item unifactorial measure of relationship satisfaction with items including, “How well does your partner meet your needs?” and “To what extent has your relationship met your original expectations?”); (b) the Passionate Love Scale (PLS; Hatfield & Sprecher, 1986), a 15‐item measure of passionate love consisting of both positive and negative cognitive, emotional, and behavioral statements such as, “Knowing that cares about me makes me feel complete,” “I would rather be with than anyone else,” “Sometimes I feel I can't control my thoughts; they are obsessively on.”]; (c) a sexual satisfaction item asking, “How happy are you with your sex life with your partner?”, on a seven‐point scale; and (d) a sexual frequency item asking “How frequently do you and your partner engage in sexual activity?”, coded as times per week.

### 2.3 Imaging data acquisition and analysis

MRI scanning was performed with a 3.0T Siemens Trio and a 12‐channel phased‐array head coil used for the acquisition of Blood Oxygenation Level Dependent (BOLD) responses. A single‐shot echo planar imaging (EPI) sequence that is sensitive to BOLD contrast was used to acquire 37 slices per repetition time (TR = 2,000 ms, 3 mm thickness, 0.5 mm gap), echo time (TE) of 30 ms, flip angle of 90 degrees, field of view (FOV) of 192 mm, and 64 × 64 acquisition matrix. Prior to the acquisition of BOLD responses, a high‐resolution T1‐weighted sagittal sequence image of the whole‐brain was obtained (TR = 15.0 ms; TE = 4.2 ms; flip angle = 9 degrees, 3D acquisition, FOV = 256 mm; slice thickness = 0.89 mm, acquisition matrix = 256 × 256). All pre and postdata processing was conducted with SPM (5 and 12). Functional EPI volumes were realigned to the first volume, smoothed with a Gaussian kernel of 6 mm, and then normalized to the T1.nii image template. Data were smoothed with a 6 mm smoothing kernel as this is a minimum standard and sufficient for subcortical regions (Hopinger, Büchel, Holmes, & Friston, 2000; White et al., 2001). No participant showed movement greater than 3 mm (whole‐voxel). After preprocessing contrasts were created for the Partner versus HFN, which were used in all further analyses.

#### 2.3.1 Multiple regression data analysis

Multiple regressions were conducted to estimate group brain activity in response to the Partner (vs. HFN), entering the sex satisfaction or frequency ratings, and genotype (either *Oxtr* or *Avpr)* in the general linear model function. The effects of *AVPR1a* rs3and *OXTR* rs53576 were tested in separate models because the impact of these polymorphisms on OT and AVP receptor function is still unclear, as OT and AVP sometimes bind to similar receptors making it difficult to discern their distinct function and binding sites (Freeman et al., 2016; Song & Albers, 2018). Thus, results are for each separate regression. There were no significant differences for sex, age, or relationship length so we proceeded with analyses not controlling for these variables. A brain response correlation, positive or negative, localizes a functional change, which is the purpose of this study. Results referred to as neural/brain activity/activation/ response herein were measured with BOLD signals.

#### 2.3.2 Region of interest (ROI) and whole‐brain analysis

ROIs were selected a‐priori and were derived from fMRI studies of early and long‐term pair‐bonds, and a meta‐analysis on sex arousal (Table 1). ROIs were explored with small volume corrections (SVCs), applying a false discovery rate (FDR) of *p* < 0.05 (to correct for multiple comparisons (Genovese, Lazar, & Nichols, 2002), and occupying a 3–10 mm radius, depending on the size of the brain area. For whole‐brain exploratory analyses, we applied a threshold of *p* ≤ 0.001 (uncorrected), with a spatial extent of ≥15 contiguous voxels. Regions were confirmed with the Atlas of the Human Brain (Mai, Paxinos, & Voss, 2008). Tables 2, 3, 4, 5 report effects replicated at T1 and T2, which were conducted to control for false positives due to small sample size. However, T1‐ and T2‐specific results are reported in Supplementary Tables S1–S4.

**Table 1:** A‐priori regions of interest

Brain region	x	y	z	Reference(s)
Ventral tegmental area/substantia nigra	±0/9	−12/24	−8/16	5,6,88
Ventral pallidum^a^	±9	6	−8	6
Nucleus accumbens	±10	4	−4/12	5
Caudate	±18	24	−2	5,6,11,87,88
Putamen	±22	2	4	5
Globus pallidus	±22	6	−8	5
Periaqueductal gray	±22	−31	−12/24	5
Thalamus	±4/12	−6/14	6/10	5,11,88
Hypothalamus	±4/6	−6	4/−12	5,11,88
Amygdala	±18/26	0	−12/20	5,88
Hippocampus	±30	−20/34	−4/18	5
Anterior cingulate	±2/6	16/36	24/36	87,88
Posterior cingulate	±6/10	−45/64	10/21	5,87
Insula	±32/44	8/14	−2/14	5,88
Inferior frontal gyrus	±50	12/28	24/34	88
Prefrontal cortex	±2/30	30/45	30	5,87,88
Angular gyrus^b^	±46	−50	26	5
Parietal/TPJ	±50	−24/42	18/33	87,88
Mid‐temporal/fusiform gyrus	±46	−50/60	−10	5,88
Occipital lobe	±46	−70/80	−8/8	88
Motor cortex	±24	−8/8	50	88

a Researched by Aron et al. (2005).

b Researched by Ortigue et al. (2007).

**Table 2:** Regional brain correlations with sexual satisfaction in response to partner versus HFN face images, replicated 1‐year apart

Brain region	Side	x	y	z	Time 1			Time 2		
					T	*p*	k	T	*p*	k
Region of interest (ROI) positive correlations										
SN, lateral^ab^	R	12	−12	−15	2.34	0.05	4	2.25	0.05	4
Globus pallidus^c^	R	15	4	−4	2.17	0.04	3	3.02	0.03	3
Hippocampus/dentate	R	33	−11	−18	3.70	0.002	7	2.68	0.02	18
Inferior frontal gyrus	R	60	18	24	3.20	0.01	6	1.90	0.03	3
Whole‐brain positive correlations										
Superior/inferior temporal gyrus^c^R		58	−18	−9	4.87	≤0.001	70	3.54	0.001	38
SI/SII	L	‐46	–18	12	4.50	≤0.001	71	4.12	≤0.001	40

Note

Superscripts indicate overlap with results for: ^a^ *Avpr* x sex satisfaction, ^b^ *Avpr* x sex frequency, and ^c^ *O*xtr x sex frequency. All *p*‐values are for voxel‐level results.

**Table 3:** Sex satisfaction × Avpr or Oxtr gene correlations with human brain response to partner versus HFN face images replicated 1‐year apart

Brain region	Side	x	y	z	Time1			Time 2		
					T	*p*	k	T	*p*	k
Sex satisfaction × Avpr brain activations										
VTA	L/R	−4	−21	−21	2.29	0.03	5	4.21	≤0.001	6
SN, lateral^ad^	R	12	−12	−12	3.02	0.02	7	2.5	0.05	4
VP^bc^	L	−8	4	−6	4.61	0.01	7	3.59	0.02	4
Hypothalamus,	L	−6	0	−5	2.96	0.02	5	3.59	0.02	5
	R	15	23	−3	2.95	0.03	5	3.21	0.03	4
Cingulate gyrus	L	−15	−18	45	4.48	0.01	7	4.27	0.004	7
Hippocampus, posterior	R	21	−33	6	2.6	0.04	3	4.64	0.003	6
Sex satisfaction × oxtr brain activations										
Hypothalamus, periventricular^bd^	L/R	0	0	−9	2.50	0.04	5	4.08	0.001	4
Accumbens/VP/hypothalamus^bc^	L	−6	3	−9	3.73	0.01	7	2.8	0.03	4
IPS	R	39	−39	33	3.04	0.01	7	3.14	<0.001	3
Dorsolateral PFC^e^	L/R	−1	42	30	3.24	0.01	7	4.32	<0.001	5
Caudate tail	R	30	−12	−9	3.86	0.02	5	3.48	0.02	4

Note

Superscripts indicate overlap with results for: ^a^sex satisfaction, ^b^ *Oxtr* × sex satisfaction, ^c^ *Avpr* × sex satisfaction, ^d^ *Avpr* × sex frequency, and ^e^ *Oxtr* × sex frequency. All *p*‐values are for voxel‐level results.

**Table 4:** Regional brain correlations with sexual frequency in response to partner versus HFN face images, replicated 1‐year apart

Brain region	Side	x	y	z	Time 1			Time 2		
					T	p	k	T	p	k
Region of interest (ROI) positive correlations										
Caudate, anterior	L	−21	18	12	2.84	0.004	29	4.38	≤0.001	30
Amygdala/para‐hippocampal gyrus		−24	2	−24	3.03	0.01	25	4.86	≤0.01	28
Insula/Piriform cortex	L	−27	15	−15	3.67	≤0.001	73	2.03	0.04	11
Dorsal ACC	R	8	32	24	3.06	0.004	68	2.77	0.01	53
Angular gyrus	R	63	−46	30	2.99	0.004	39	2.24	0.05	5
Parietal operculum/SII	R	48	−24	21	2.32	0.02	29	4.31	≤0.001	95
Mid temporal gyrus/FFA^a^	L	−48	−63	4	3.47	≤0.001	61	2.56	0.02	34
Pre/motor cortex	L/R	−0.61538	3	58	2.39	0.05	11	2.34	0.02	4
Whole‐brain positive correlations										
Precentral gyrus	R	63	−12	27	4.19	≤0.001	50	3.04	0.001	64
Mid temporal gyrusa	L	−57	−15	−9	4.18	≤0.001	97	4.21	0.001	20

Note

Superscript indicates overlap with results for ^a^ *Oxtr* × sex frequency.

**Table 5:** Sex frequency × Avpr and Oxtr gene correlations with human brain response to partner versus HFN face images replicated 1‐year apart

Brain region	Side	x	y	z	Time 1			Time 2		
					T	*p*	k	T	*p*	k
Sex frequency × Avpr brain activations										
SNabd	R	12	−12	−18	1.83	0.05	4	2.6	0.05	5
Hypothalamus/paraventricular^bc^	L	−5	0	−6	2.05	0.03	4	3.63	0.03	4
Sex frequency × Oxtr brain activations										
GP^a^	R	12	3	0	2.94	0.01	7	2.46	0.05	7
Putamen	R	24	0	6	2.51	0.04^a^	4	5.08	≤0.001	6
Anterior cingulate	R	5	17	35	7.21	≤0.001	7	4.86	≤0.001	17
Inferior temporal gyrusa	R	45	−9	−27	2.85	0.002^a^	4	4.96	≤0.001	4
Mid temporal gyrus^d^	L	−57	−9	−9	4.04	≤0.001	5	3.23	0.03	3
Dorsolateral PFC^d^	L/R	−1.11429	39	30	2.01	0.04^a^	7	3.01	0.01	7

Note

Superscripts indicate overlap with results for: ^a^sex satisfaction, ^b^ *Avpr* × sex satisfaction, ^c^ *Oxtr* × sex satisfaction, and ^d^sex frequency. All *p*‐values are for voxel‐level results.

## 3 RESULTS

### 3.1 Behavioral findings

#### 3.1.1 Relationship ratings

Participants reported relatively high levels of sexual satisfaction (T1: *M* = 5.90, *SD* = 1.13, range = 3.0–7.0; T2: *M* = 5.23, *SD* = 1.54, range = 1.0–7.0); weekly sexual activity (T1: *M* = 3.3, *SD* = 2.2, range = 0.80–7.0; T2: *M* = 1.83, *SD* = 1.25, range = 0.30–5.0), PLS (T1: *M* = 5.9, *SD* = 0.7, range = 4.1 – 6.9; T2: *M* = 5.7, *SD* = 1.0, range = 3.0–6.7); and relationship satisfaction scores on the RAS (T1: *M* = 6.35, *SD* = 0.59, range = 5.0–7.0; T2: *M* = 6.34, *SD* = 0.56, range = 5.0–7.0). Repeated measures *t* tests showed no significant differences across time (*p* > 0.05, two‐tailed) for relationship ratings. Correlations between the PLS and sex satisfaction and frequency were not significant (*r*'s = 0.06 to 0.45; *p*'s > 0.05), except for PLS with sex satisfaction at T2 (*r* = 0.60, *p* ≤ 0.05).

#### 3.1.2 Attractiveness ratings of photos by independent raters

Attractiveness ratings of opposite‐sex face images were provided by six coders (three females) recruited for this task, showing adequate inter‐rater reliability [at T1: females (α = 0.71), males (α = 0.84); and T2 females (α = 0.62), males (α = 0.82)]. Ratings showed no objective significant differences in facial attractiveness for: T1 Partner (*M* = 4.76, *SD* = 1.98) vs. T1 HFN images (*M* = 4.13, *SD* = 1.40), *t*(17) = 1.40, *p* > 0.05; and T2 Partner (*M* = 5.31, *SD* = 1.11) vs. T2 HFN images (*M* = 4.56, *SD* = 0.85), *t*(12) = 1.76, *p* > 0.05.

### 3.2 fMRI results

#### 3.2.1 Neural correlates of sexual satisfaction at T1 and T2

**Figure 1:**
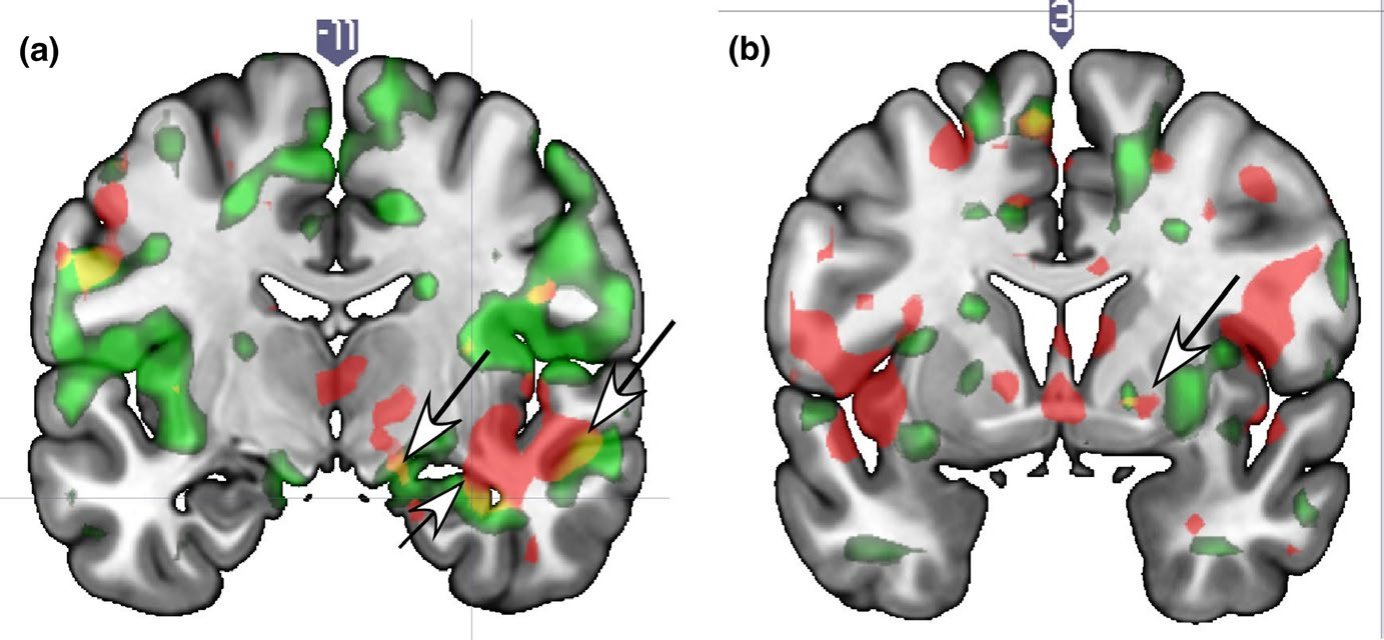
Brain localizations showing positive correlations with sexual satisfaction scores in response to viewing face images of a romantic partner versus a familiar acquaintance at T1 and T2. (a) The lateral substantia nigra (top left arrow), hippocampal region (lower middle arrow) and superior/inferior temporal sulcus (top right arrow) shown for at both T1 and T2. (b) The right globus pallidus shows a correlation for sexual satisfaction at both time points (arrow) and there is an overlapping region. Legend. Red = T1 only; Green = T2 only; Yellow = overlap of T1 and T2

ROI analysis showed that sexual satisfaction was positively associated with neural activity in the right far lateral SN (Figure 1a), a small area of the globus pallidus (GP) (Figure 1b), the hippocampus/dentate/ amygdala (Figure 1b); and the inferior frontal gyrus sh(IFG) (Table 2) at both T1 and T2. Whole‐brain results showed significant correlations in the right superior/inferior temporal gyrus (STG, ITG), and the secondary somatosensory area (SII).

#### 3.2.2 Neural correlates of sexual satisfaction specific to T1 or T2

T1‐specific correlations of sexual satisfaction were shown in the VTA, accumbens, caudate body, hypothalamus, bed nucleus of the stria terminalis (BNST), thalamus, cingulate, AG, ventrolateral and dorsomedial PFC, SFG, and premotor areas. T2‐specific results showed positive correlations with sex satisfaction scores in the ventral pallidum (VP), raphe, pons, caudate tail, occipital gyrus, and SII (Supplementary Table S1).

#### 3.2.3 Neural correlates of sexual satisfaction x *Avpr* at T1 and T2

**Figure 2:**
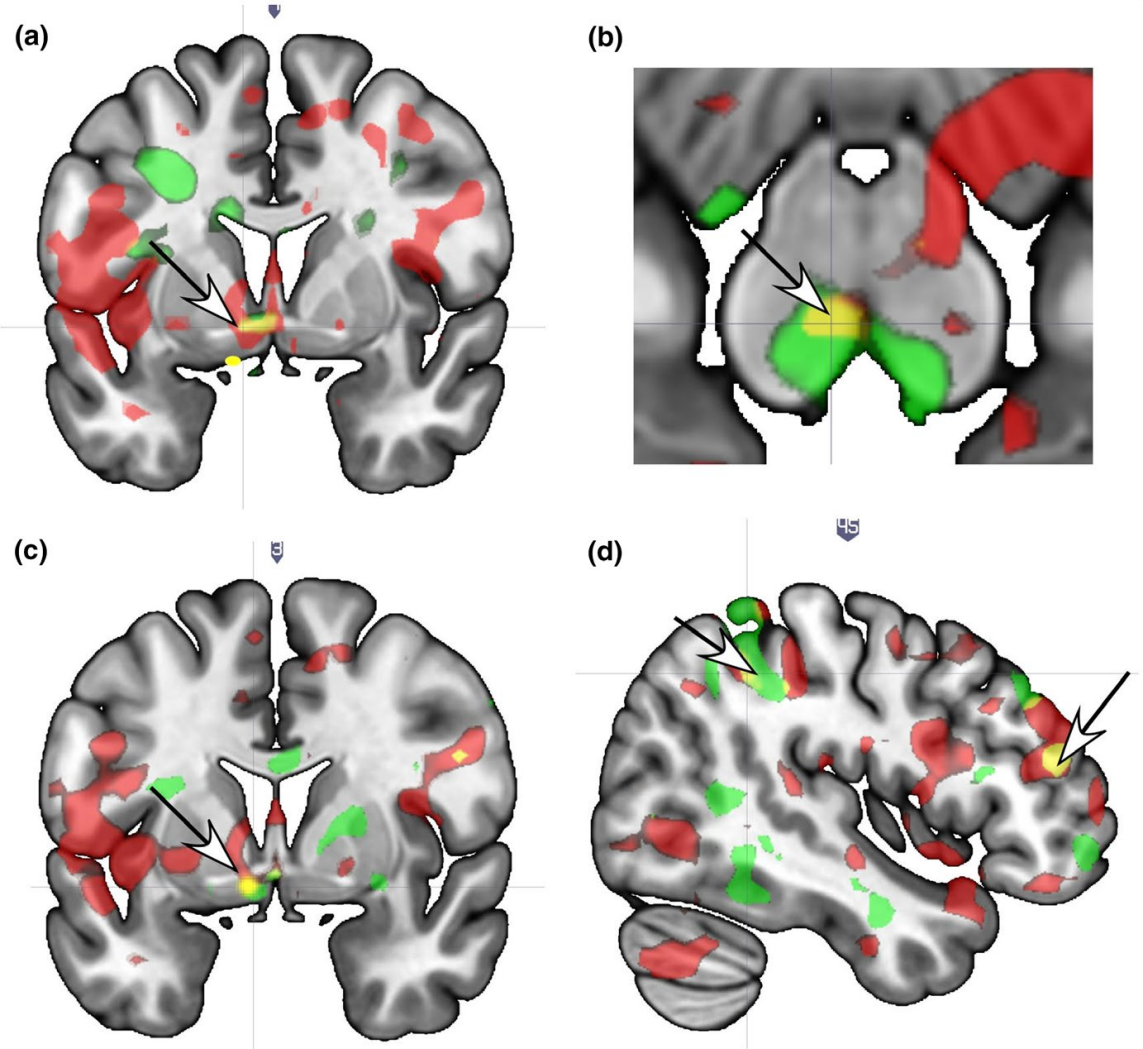
Brain localizations showing significant response interactions with sexual satisfaction scores and *OXTR* and/or *AVPR*. (a) The left ventral pallidum shows an interaction with *AVPR* at both T1 and T2 (arrow). (b) The left VTA shows an interaction with *AVPR* at both T1 and T2 (arrow). (c) The left ventral pallidum/anterior hypothalamus regions show an interaction with *OXTR* (arrow). (d) The dorsolateral prefrontal cortex (right arrow) and the intraparietal sulcus region (left arrow) show an interaction with *OXTR* at both T1 and T2. Legend. Red = T1 only; Green = T2 only; Yellow = overlap of T1 and T2

*Avpr* long alleles were associated with greater sexual satisfaction scores in the left VP (Figure 2a); bilateral VTA (left area covered greater than right) (Figure 2b); right SN, caudate head, posterior hippocampus; and the left paraventricular hypothalamus and cingulate gyrus (CG) at both T1 and T2 (Table 3). The left VTA and VP responses in association with greater number of *Avpr* long alleles were particularly robust at T2 (*r* = 0.76, *p* < 0.01; *r* = 0.58, *p* < 0.05, respectively).

#### 3.2.4 Neural correlates of sexual satisfaction x *Avpr* specific to T1 or T2

At T1, the interaction of *Avpr* (long alleles) and sexual satisfaction (greater) showed positive associations in the right entorhinal area, amygdala; left insula, IFG, orbitofrontal gyrus (OFG), SFG, IPS, parietal area, SMA; and bilateral dorsolateral PFC. T2‐specific interactions of *Avpr* (long alleles) with sexual satisfaction scores showed positive associations in the bilateral hippocampus; left amygdala/entorhinal cortex, caudate tail, inferior colliculus, frontal sulcus, and MFG; and the right cingulate/OFG and insula (Supplementary Table S2).

#### 3.2.5 Neural correlates of sexual satisfaction x *Oxtr* at T1 and T2

Results showing significant interaction effects for *Oxtr* (G alleles) at both T1 and T2 with sex satisfaction were seen in the left accumbens, VP and hypothalamus (Figure 2c); as well as the right IPS and bilateral DLPFC (Figure 2d), paraventricular hypothalamus, and the right caudate tail (Table 3).

#### 3.2.6 Neural correlates of sexual satisfaction x *Oxtr* specific to T1 or T2

Sex satisfaction x *Oxtr* (G alleles) interactions resulted in T1‐specific localizations in the right VTA/SN, entorhinal area/ hippocampus, STG; bilateral posterior cingulate; left putamen, mid‐insula, and SFG. T2‐specific responses were shown in the left VTA/SN, pons/PAG; bilaterally in the GP; and the right amygdala, lingual gyrs, and temporal/auditory cortex (Supplementary Table S2).

#### 3.2.7 **Neural correlates of sexual frequency a**t **T1 and T2**

**Figure 3:**
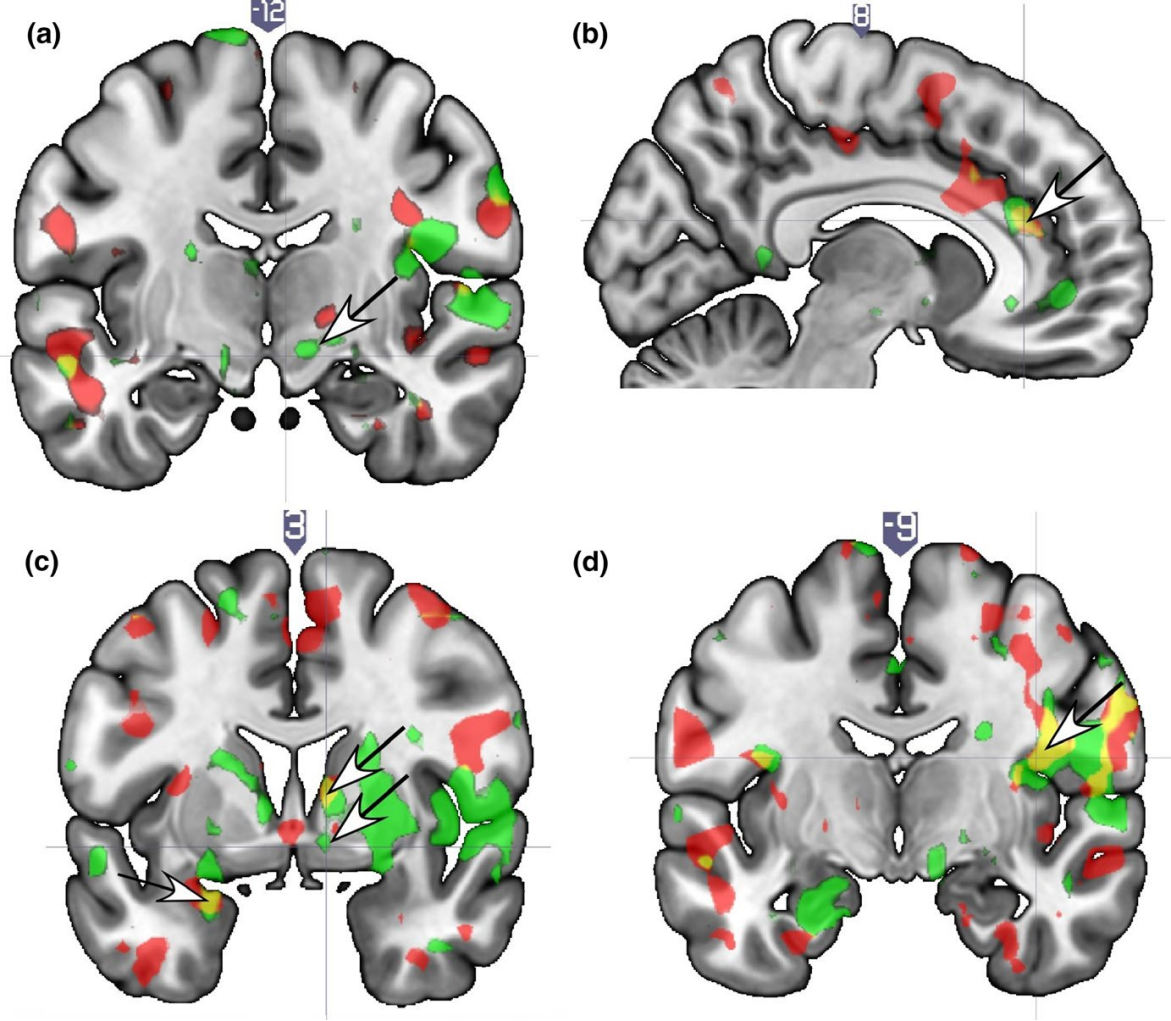
Brain localizations showing positive correlations with self‐reported sex frequency (times/week) while viewing face images of a spouse versus a familiar acquaintance. (a) The substantia nigra region is not correlated with sexual frequency at both time points, as it is for sexual satisfaction (arrow; compare to Figure 1a). (b) Anterior cingulate cortex (arrow). (c) Amygdala (arrow). (d) Parietal operculum/SII. Legend. Red = T1; Green = T2; Yellow/orange = overlap of T1 and T2

Sexual frequency was positively correlated with activation of the right dorsal ACC (Figure 3b; left caudate anterior, amygdala parahippocampal gyrus (Figure 3c), insula/ piriform cortex, MTG/fusiform gyrus; angular gyrus; parietal operculum/SII (Figure 3d); and the bilateral pre/motor cortex (ROIs); as well as the right precentral gyrus and left MTG (whole‐brain), at both T1 and T2 (Table 4).

#### 3.2.8 Neural correlates of sexual frequency specific to T1 or T2

Neural correlates of sexual frequency specific to T1 were seen in the VP/accumbens (Figure 3c) (which was in the same region that correlated with sexual satisfaction, compare Figures 1c and 3c) cingulate, IFG, SFG, STG, temporal pole, inferior parietal cortex, parietal operculum/SII, and SMA. T2‐specific activations for sex frequency were seen in the SN, putamen, GP, mid‐insula, medial PFC, and dorsolateral PFC (DLPFC) (Supplementary Table S3).

#### 3.2.9 Neural correlates of sexual frequency x *Avpr* at T1 and T2

**Figure 4:**
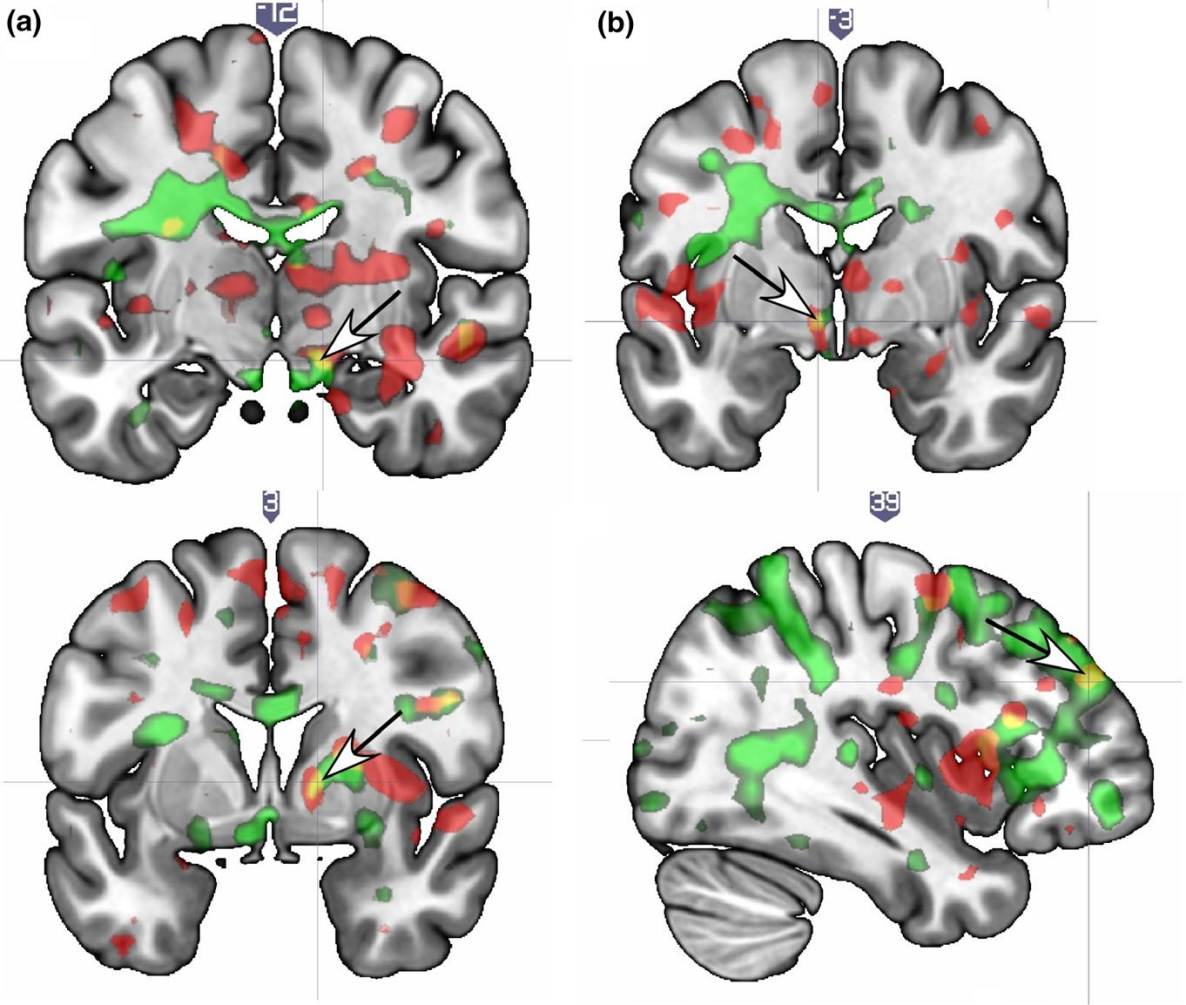
Brain localizations showing significant brain response interactions with sex frequency (times/week) and *OXTR* or *AVPR*. (a) At both T1 and T2 the substantia nigra region shows an interaction for *AVPR* (arrow) and sex frequency, which is the same region that correlated with sexual satisfaction (see Figure 1a). (b) The hypothalamus/paraventricular region showed an *AVPR* interaction at T1 and T2 (arrow). (c) The globus pallidus shows an interaction with OXTR at T1 and T2 (arrow). (d) The dorsolateral prefrontal cortex shows an interaction with OXTR at T1 and T2 (arrow). Legend. OXTR = Red; AVPR = Green

*Avpr* (greater number of long alleles) and sex frequency showed significant interaction effects at T1 and T2 in the right SN (Figure 4a), where sexual satisfaction also showed an effect at T1 and T2 (compare Figures 1a and 4a), and the left hypothalamus/paraventricular region (Figure 4b and Table 5).

#### 3.2.10 Neural correlates of sexual frequency x *Avpr* specific to T1 or T2

T1‐specific neural effects for *Avpr* × sexual frequency were shown in the VP (bilaterally), caudate, hippocampus, cingulate, IFG, operculum, AG, MTG, STG, and the PFC. (Supplementary Table S4). T2‐specific effects for the *Avpr* x sexual frequency interaction were shown in the VTA/SN, caudate, cingulate, inferior colliculus, thalamus, amygdala, hippocampus, AI, MFG, orbitofrontal gyrus (OFG), IPS, and SII. At T2, robust correlations were shown for *Avpr* and sexual frequency in the lateral SN (*r* = 0.60, *p* < 0.05), and the amygdala (*r* = −0.6, *p* < 0.05).

#### 3.2.11 Neural correlates of sexual frequency x *Oxtr* at both T1 and T2

*OXTR* (greater number of G alleles) × greater sex frequency/week showed positive associations in the right GP (Figure 4c), bilateral DLPFC (Figure 4d); putamen, AC, ITG, and the left MTG (whole‐brain) (Table 5).

#### 3.2.12 Neural correlates of sexual frequency x *Oxtr* specific to T1 or T2

T1‐specific neural effects for the *Oxtr* × sex frequency interaction were shown within the parahippocampal gyrus, insula, IFG, and MFG. T2‐specific effects for the *Oxtr* × sex frequency interaction were shown in the cingulate gyrus, AC, thalamus, hippocampus/entorhinal area, hypothalamus, MFG, IPS, and premotor cortex/caudate (Supplementary Table S4).

### 3.3 Genotype distributions

**Figure 5:**
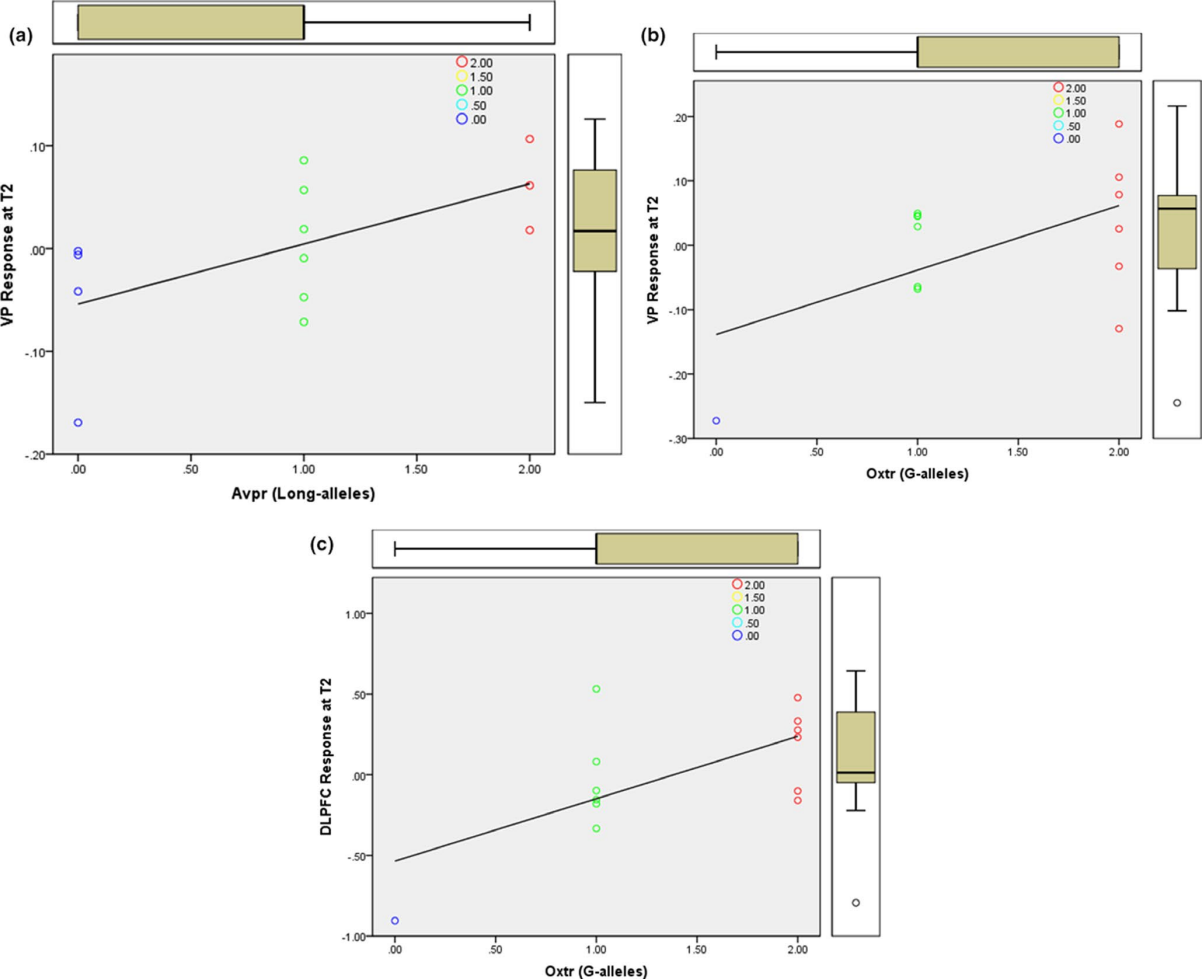
(a) Scatterplot shows the T2 correlation between Avpr1a rs3 (long‐alleles) and ventral pallidum (VP) response. (b) Scatterplot shows the T2 correlation between Oxtr rs53576 genotype with VP response. (c) Scatterplot shows the T2 correlation between Oxtr rs53576 genotype with response in the DLPFC

Genotype distributions for the sample were as follows. For *Oxtr rs53576* (AA = 1, AG = 6, GG = 6). For *Avpr1a rs3* (short = 4, short/long = 6; long = 3). Scatterplots show the T2 correlations between *Avpr1a rs3* and ventral pallidum (VP) response (Figure 5a), and *Oxtr rs53576* genotype with VP response (Figure 5b) as well as the dorsolateral PFC (Figure 5c).

## 4 DISCUSSION

This study was the first to examine the neural correlates of sexual satisfaction and frequency, and their interaction with *Oxtr* and *Avpr* gene variants among pair‐bonded individuals that were scanned twice, over 1‐year, while viewing face images of a partner. Results showed that satisfying sexual activity conferred strong activation in brain regions involved in reward and pair‐bonding (the SN, VTA, caudate, and VP/GP) emotion and memory (amygdala and hippocampus); hormone balance (hypothalamus), executive/behavioral control (DLFC), and self‐other, reflective, and sensory processing (IFG, AG, temporal gyri, IPS, SI/SII/parietal lobes). These data provide robust evidence of the neural correlates of sex satisfaction in pair‐bonds, as our main results were replicated with fMRI scans of newlywed individuals over the first year of marriage.

In line with animal models of pair‐bonding (Lim & Young, 2004; Young, Huot, Nilsen, Wang, & Insel, 1996; Young & Wang, 2014), the current sample showed significant activation in regions that are rich in receptors for OT, AVP, and opiates—which modulate reward, attention, emotion/memory, and hormones. Moreover, the pattern of results was amplified for individuals with *Oxtr* (G‐alleles) and *Avpr* (long‐alleles) variants associated with complex social behaviors, including pair‐bonding, and altruism. For humans, however, mating processes appear to be more complex as reflected by activation of cortical networks that support high‐order thinking, planning, and behavioral control; as well as self‐other, and reflective processes. Thus, the present findings are consistent with theoretical models of sexuality in humans that emphasize both conscious, effortful, and deliberate processes; and automatic, unconscious processes (Stoléru et al., 2012). They also expand models of human mating by showing that beyond reproduction, satisfying sexuality may facilitate neural activity associated with reward, well‐being, and intimacy in pair‐bonds. Thus, building on the current body of work on the neural basis of sex, this study showed with partner‐specific stimuli (in contrast to studies of sex arousal which have almost solely relied on general sexual stimuli and pornography), the neural and genetic correlates of a psychological aspect of sex—perceived sex satisfaction with a marital partner.

### 4.1 Sex, reward, and well‐being

It is interesting to note that both sex satisfaction and frequency (and their interactions with *Oxtr* and *Avpr*) showed significant correlations with neural activity in reward centers, such as the VTA, SN, caudate, and the VP/GP. These regions are rich in binding sites for dopamine, oxytocin, and vasopressin receptors (Freeman, 2017), and they have been shown in over a dozen studies of romantic love (Aron et al., 2005; Acevedo et al., 2012; Bartels & Zeki, 2000; Bartels & Zeki, 2004; Xu et al., 2012) and pair‐bonding in nonhuman monogamous mammals (Donnelly, 1993; Lim & Young, 2004; Young & Wang, 2014; Yucel & Gassanov, 2010). The VTA/SN are major dopamine‐sites, whose neurons mediate approach motivation, euphoric experiences, sexual arousal, and response to novel, rewarding, and addictive stimuli (Berridge & Robinson, 2003; Childress et al., 2008; Georgiadis et al., 2010; Ikemoto, Yang, & Tan, 2015; Noori, Cosa, & Spanagel, 2016; Schultz, 2010; Stoléru et al., 2012).

The VP was a key ROI, as direct injections of OT and AVP into the brains of voles showed that OT and AVP receptor binding within the VP was critical for establishing partner preference after mating (Lim & Young, 2004). OT has long been associated with mating in mammals (Carter, 1992). Increased OT has been linked with semen emission in males, uterine contraction during female orgasm (Vignozzi et al., 2008), and plasma OT levels increase during sex and after orgasm in both men and women (Carmichael, Warburton, Dixen, & Davidson, 1994). Intranasal OT has also been associated with increased orgasm intensity, and complex social behaviors such as trust, eye contact, and increased partner empathy during sex (Behnia et al., 2014; Guastella, Mitchell, & Dadds, 2008; Kosfeld et al., 2005). Interestingly, research on sexual arousal, reported that the VP showed the highest activation during the onset of penile erection (Georgiadis et al., 2010), and even in response to subliminal sexual and cocaine cues (Childress et al., 2008). Somewhat similarly, one study showed that activation of the left nucleus accumbens (in an area close to where *Oxtr* and *Avpr* × sex satisfaction showed significant activity in this study) in response to sexual images predicted a stronger desire for partnered sex, 6 months after (Demos, 2012).

Also, with respect to the gene variants examined herein, one study showed that males with a long repeat‐sequence of the *Avpr1a rs3* gene were more likely to be married, have better partner‐bonding, and marital quality; and this was even confirmed with the spouses’ perceptions of marital quality (Walum, 2008). Studies with the *Oxtr* variant have shown that individuals with a greater number of G (vs. A) alleles display greater empathy, altruism, sensitive parenting, and sociality (Acevedo, Poulin & Brown, 2019; Buffone & Poulin, 2014; Li et al., 2015; Poulin et al., 2012; Rodrigues et al., 2009; Uzefovsky, et al., 2015). Collectively, these results lend support to models proposing that sustained sexual satisfaction within pair‐bonds, beyond reproduction, may have partly evolved for relationship‐maintenance purposes (Hicks et al., 2016; Peterson et al., 2011). This seems like an efficient evolutionary adaptation, as the pair‐bond serves multiple functions from companionship and emotional intimacy, social support, care‐giving, sex, and romantic love (Acevedo et al., 2014; Collins & Feeney, 2000).

However, as with other traits, humans show diverse strategies to increase overall fitness of the species (Boyce & Ellis, 2005). Consistent with these views, herein we showed that the strength of activation in brain structures that modulate reward and bonding behaviors (VP, VTA, and SN), in response to a partner's face image, varied as a function of *Avpr* and *Oxtr* genotypes, perhaps explaining variation in the expression of pair‐bonding and mating strategies. The present findings also lend some clarity to previous research showing that greater sexual frequency was associated with enhanced relationship satisfaction in only *some* couples (Brody et al., 2018; Loewenstein et al., 2015; McNulty et al., 2016; Schoenfeld et al., 2017). Thus, we suggest that behavioral changes (i.e., increases in sexual frequency) may result in particularly strong benefits for individuals/couples with the *Oxtr* and *Avpr* genotype variants associated with pair‐bonding and complex social behaviors.

The present findings highlight some of the neural processes that mediate the link between satisfying sex acts with relationship and individual well‐being. For example, across various conditions, significant activations were shown in the amygdala, hippocampus, and hypothalamus—brain structures that are classically known for their involvement in the processing of emotions, memory, hormones, and sexual arousal (Brunetti et al., 2008; Davis & Whalen, 2001; Curtis & Pare, 2004; Ferretti et al., 2005; Karama et al., 2002; Pfaus, 1999). In fact, some theories suggest emotion as a cognitive component of sex (Stoléru et al., 2012), which may serve as a precursor to sexual arousal or a modulator of emotions, through the release of hormones involved in stress‐relief and calm (Brody, 2006).

It is also interesting that hippocampal activation was stronger in response to face images of a partner for those reporting more satisfying sex (and also as a function of the *Oxtr*), as this was also shown in a previous study examining individuals in long‐term marriages of about 20 years on average (Acevedo et al., 2012). Also, animal studies have shown that sexual activity promotes hippocampal and cognitive functioning in rats (Glasper & Gould, 2013). Correspondignly, a review of eight studies on sexual activity in healthy and demented samples showed that healthy individuals that continued to engage in sexual activity had better overall cognitive functioning. Also, cognitive decline and dementia were associated with diminished sexual behavior in older persons (Hartmans, Comijs, & Jonker, 2014). It is also noteworthy that the present sample showed activation of the hypothalamus in association with both sexual satisfaction and frequency as one study with middle‐aged males (ages 46–55), failed to show hypothalamic response to erotic films (Kim et al., 2006). Perhaps these discrepancies may be accounted for by the type of stimuli used, such that individuals in pair‐bonded relationships may show differential patterns of neural activation to their own or potential mating partners, and also by the individual's genotype.

### 4.2 Sex, intimacy, and the human brain

The present results also extend current models of pair‐bonding by showing that in humans, satisfying sex acts evoke activation in brain structures such as the insula, IFG, AG, parietal, and temporal areas. These regions coordinate higher‐order cognitive processes including empathy, meaning‐making, awareness, and self‐other processes (Arzy, Thut, Mohr, Michel, & Blanke, 2006; Brewer et al., 2011; Cabeza & Nyberg, 2000; Cauda et al., 2012; Hein & Knight, 2008; Jabbi, Bastiaansen, & Keysers, 2008; Lamm, Decety, & Singer, 2011; Lauwereyns, Watanabe, Coe, & Hikosaka, 2002; Olson, McCoy, Klobusicky, & Ross, 2013; Ortigue, Bianchi‐Demicheli, Hamilton, & Grafton, 2007; Singer et al., 2004; Tel et al., 2002). It is interesting to note that these areas are not typically shown in sex studies with nonhumans. However, their activation in the context of in‐pair copulation is consistent with dual‐process models of relationships which propose that in ancestral species, partner‐preference processes evolved before the capacity for complex deliberative reasoning existed in humans. Thus, the newer and more complex cognitive deliberative‐reasoning processes allow humans to override their automatic inclinations, and to make explicit judgments and decisions regarding partner selection and relationship processes (Fazio & Olson, 2014). With respect to sex, as well, links with higher‐order cortical systems mediate the more complex processes observed in human sexual pair‐bonds such as intimacy, trust, closeness, and partnerships that may or may not include offspring. Thus, activation of these phylogenetic newer areas (as well as their associations with *Oxtr* and *Avpr* variants) may explain the flexibility in mating strategies observed in humans (Geher & Kaufman, 2014; Peterson et al., 2011).

These data highlight how positive sex acts in human pair‐bonds, beyond reproduction, are associated with neural processes that modulate complex psychological phenomenon, such as meaning‐making, perspective‐taking, cognitive/emotional intimacy, and closeness (Acevedo, 2017; Aron & Mashek, 2013); and perhaps even more importantly the deliberate, self‐regulatory processes necessary to initiate and sustain pair‐bonds. The pattern of overall results suggests that positive and frequent sex acts in couples are associated with activation of neural regions involved in reward, hormone‐control, attention, and self‐other, and self‐regulatory processes. These circuits also mediate processes related to mood, sleep, and physiological homeostasis (Olson et al., 2013). In sum, sex acts may directly increase fitness by affecting neurochemicals involved in physiological and psychological homeostasis; and indirectly by promoting pair‐bond quality and stability to ensure love, care, and meaningful experiences for the coupled partners. In sum, we extend current models of human mating and pair‐bonding, highlighting the importance of both basic reward processes and attachment, as well as higher order cognitive processes that mediate self‐control and reflective‐processing and self‐other merger.

### 4.3 Limitations and future directions

This is the first study to examine the neural and genetic correlates of sexual satisfaction and frequency among pair‐bonded individuals in response to a partner's face image. We showed that both subcortical and cortical systems are significantly associated with positive sex acts in pair‐boned humans, and to a greater extent for individuals with *Oxtr* and *Avpr* variants associated with social behaviors. This study, in addition to contributing to the basic biological knowledge on sex and pair‐bonding in humans, also provides a basis for basic applications as it highlights the importance of intimacy processes (and their neural correlates) with respect to satisfying sex acts. However, as with any study, there are limitations. For example, the small sample size (18 individuals total) and lack of a control group remain to be addressed in future studies. However, these issues were mitigated, to some extent, by our emphasis of only effects that were replicated at two scans (conducted one‐year apart). Also, this is a major strength as experts of fMRI research advocating for the reproducibility of fMRI results have suggested that smaller, more constrained samples may produce more consistent effects, as larger sample sizes do not always result in consistent replications. Nevertheless, it will be important to replicate these findings in more diverse and larger samples. Also, future studies may wish to use more varied, multi‐item measures of sex satisfaction as well as examining other types of partner stimuli (such as voices or smell). Also they may wish to examine these processes in couples experiencing sexual and/or relationship issues, and with network analysis and PET for more direct evidence of *Oxtr's* and *Avpr's* effects. Nevertheless, the present results are novel as they examine basic biological substrates for satisfying sexual activity, a central factor in pair‐bond initiation and maintenance (Donnelly, 1993; Lim & Young, 2004). They extend our understanding of this basic human motivation, sexual mating with a partner, and provide support for relationship and biological models of pair‐bonding.

## 5 CONCLUSIONS

Sexuality in humans is complex and it includes a variety of activities that are not obviously and directly relevant to reproductive success. We found that a positively perceived sex life among pair‐bonded individuals recruits a suite of brain regions associated with reward, emotion, attention, memory, physiological homeostasis; and complex cognitive processing suggesting self‐other integration and empathy. Interestingly reward and hormone‐control effects were stronger for individuals with *Oxtr* and *Avpr* genotypes associated with complex social and pair‐bonding behaviors. We conclude that beyond reproduction, a satisfying sex life is a healthy and, rewarding attachment variable that may support individual and relationship well‐being; and to a greater extent for individuals with *Oxtr* and *Avpr* genotype variants associated with complex social behaviors.

## CONFLICT OF INTEREST

The authors report no competing interests or conflict of interest to report.
