# Supplementary material for: The neural and genetic correlates of satisfying sexual activity in heterosexual pair‐bonds
Source: Brain Behav. 2019 May 14;9(6):e01289. doi: 10.1002/brb3.1289 (PMC6576152; doi:10.1002/brb3.1289)
Supplement: Supplementary file 1 [file BRB3-9-e01289-s001.pdf]

Supplementary Table 1. ***Regional Brain Correlations with Sexual Satisfaction Scores in Response to Partner Face vs. HFN Face Images at T1 or T2***

Brain Region	Side	x	y	z	T	*p*	k
**Time 1**							
VTA	R	6	-6	-12	2.26	0.03	5
Accumbens	R	13	13	-4	2.58	0.01	7
Caudate body	R	12	-3	18	2.54	0.01	5
Globus pallidus	R	18	4	-5	2.58	0.05	3
Amygdala	R	18	-6	-16	2.20	0.05	3
Hypothalamus	R	6	-6	-9	2.28	0.01	5
BNST	L/R	-1/2	3	-2	3.28	0.04	7
ACC	L/R	-3/8	40	12	3.52	0.01	7
Thalamus	L/R	-3/5	-12	9	2.24	0.04	5
IFG	L	-60	15	24	2.56	0.04	7
AG	L/R	-51/57	-48	33	3.57	0.05	7
Ventrolateral PFC	R	45	33	-15	2.62	0.02	5
Dorsomedial PFC	L	-3	57	30	2.58	0.03	4
Medial PFC	R	6	54	3	2.42	0.04	3
Medial SFG/cingulate	L	-3	33	30	4.87	< .001	7
Pre/motor cortex	L/R	-27/45	3	51	3.04	0.004	7
**Time 2**							
VP	L	-12	6	-6	3.02	0.03	3
Raphé, reticular	R	6	-14	-10	2.25	0.01	7
Pons, dorsal	L	-15	-33	-42	2.77	< .001	7
Caudate tail	L	-33	-36	5	2.04	0.001	1
Occipital gyrus	R	027	-54	12	3.49	< .001	23
SII	L	-36	-3	18	1.84	< .001	2

HFN= Highly familiar neutral.

Supplementary Table 2. ***Sex Satisfaction x Avpr and Oxtr Gene Correlations with Human Brain Response to Partner vs HFN Face Images shown at T1 or T2***

Brain Region	Side	x	y	z	T	*p*	k
			Sex Satisfaction x Avpr				
**Time 1**							
Entorhinal area/hippocampus	R	33	-14	-20	3.40	0.01	5
Amygdala	R	30	-3	-18	3.07	0.02	6
Insula	L	-48	0	6	7.08	≤ .001	7
Inferior frontal gyrus	L	-27	30	-9	5.27	≤ .001	7
Orbitofrontal gyrus	L	-24	36	-15	4.19	≤ .001	7
SFG	L	-15	57	-3	4.21	≤ .001	5
IPS	L	-15	-63	60	4.34	≤ .001	6
Inferior parietal	L	-63	-24	21	7.56	≤ .001	7
SMA	L	-6	0	60	4.39	≤ .001	7
Dorsolateral PFC	L/R	42	42	21	4.21	0.01	7
**Time 2**							
Amygdala/entorhinal cortex	L	-6	0	-18	4.02	≤ .001	5
Hippocampus, posterior	L/R	18	-21	-9	6.97	≤ .001	6
Caudate tail	L	-18	-36	18	7.00	≤ .001	7
Inferior colliculus	L	-3	-33	-15	3.42	≤ .001	6
Cingulate/orbitofrontal gyrus	R	21	33	0	5.08	≤ .001	7
Insula, posterior	R	27	-45	27	6.54	≤ .001	7
Frontal sulcus	L	-30	3	39	6.25	≤ .001	7
Medial frontal gyrus	L	-12	45	24	5.98	≤ .001	7

Sex Satisfaction x Oxtr							
**Time 1**							
VTA/SN	R	6	-15	-12	3.30	0.004	6
Entorhinal area/hippocampus	R	33	-12	-21	3.25	0.01	4
Putamen	L	-24	3	0	4.13	0.01	7
Thalamus	L	-24	-24	6	7.16	≤ .001	6
Posterior cingulate	L/R	0/9	-30	39/45	5.53	≤ .001	7
Mid-insula	L	-42	6	-6	6.70	≤ .001	7
SFG	L	-15	57	-3	4.44	≤ .001	5
Superior temporal gyrus	R	48	-15	-12	4.43	0.01	7
**Time 2**							
VTA/SN	L	-4	-18	-15	2.11	0.05[a]	3
GP, posterior	L/R	24	-3	-6	3.20	0.02	4
Pons/PAG	L	6	-33	-27	4.39	≤ .001	5
Amygdala, central	R	26	-3	-12	3.05	0.04	4
Lingual gyrus, BA18	R	3	-63	0	5.54	≤ .001	7
Temporal gyrus/auditory cortex	R	51	-24	3	4.58	≤ .001	6

HFN= Highly familiar neutral.

Supplementary Table 3. ***Regional Brain Correlations with Sexual Frequency Shown in Response to Partner vs. HFN Face Images at T1 or T2***

Brain Region	Side	x	y	z	T	*p*	k
**Time 1**							
Cingulate gyrus	R	6	-21	42	3.52	≤ .001	7
Inferior frontal gyrus	L	-36	30	-3	3.14	0.01	6
Superior frontal gyrus	R	18	27	42	4.69	≤ .001	7
Superior temporal gyrus	L	-60	-39	12	4.96	≤ .001	7
Temporal pole	L	-42	10	-35	3.08	≤ .001	7
Inferior parietal cortex	L	-27	-39	54	3.76	≤ .001	7
Parietal operculum/SII	L	-48	-27	12	4.70	≤ .001	7
SMA	R	3	3	54	3.81	≤ .001	7
**Time 2**							
Substantia nigra	R	10	-10	-12	2.41	0.04	8
Putamen	R	24	6	12	6.13	≤ .001	6
VP/accumbens	R	8	4	-5	2.63	0.03	5
GP, posterior	R	21	0	-6	2.70	0.03	4
Mid-insula	R	44	3	3	4.96	0.01	18
Medial PFC	R/L	0	54	3	3.01	0.01	5
Dorsolateral PFC	L	-48	36	27	3.83	≤ .001	6

HFN= Highly familiar neutral.

Supplementary Table 4. ***Sex Frequency x Oxtr and Avpr Gene Correlations with Human Brain Response to a Partner vs. HFN Face Images shown at T1 or T2***

Brain Region	Side	x	y	z	T	*p*	k
Sex Frequency x Oxtr							
**Time 1**							
VP	L/R	-15	-3	-3	2.77	0.05[a]	4
Caudate, head	L	-15	19	13	3.29	0.04[a]	3
Hippocampus	R	27	-12	-30	3.38	$\leq$ .001	4
Cingulate	R	6	-18	45	4.99	$\leq$ .001	5
IFG/frontal operculum	L	-57	12	12	4.81	$\leq$ .001	7
Angular gyrus	R	45	-69	18	4.58	$\leq$ .001	7
Middle temporal gyrus	R	42	-15	-12	4.77	$\leq$ .001	7
Superior temporal gyrus	L	-30	-30	15	5.42	$\leq$ .001	7
Prefrontal cortex	L	-30	42	24	5.85	$\leq$ .001	7
**Time 2**							
Thalamus	R	21	-27	9	4.16	$\leq$ .001	6
Hippocampus/entorhinal area	R	27	-9	-12	3.44	0.003	6
Hypothalamus/BNST	L/R	-6	3	-6	4.14	0.001	5
Middle frontal gyrus	R	42	33	42	2.75	$\leq$ .001	7
IPS	R	48	-45	60	4.37	$\leq$ .001	6
Premotor cortex/caudate	R	24	-12	33	6.39	$\leq$ .001	4

Sex Frequency x Avpr							
**Time 1**							
Parahippocampal gyrus	L/R	-27/42	-24	-27	4.95	≤ .001	7
Insula	L	-30	-30	15	6.13	≤ .001	7
Inferior frontal gyrus	L/R	-33/39	15	-15	4.74	≤ .001	7
Middle frontal gyrus	R	27	-3	60	8.47	≤ .001	7
**Time2**							
VTA/SN	L/R	-6/6	-15	-18	4.92	≤ .001	6
Caudate, head	L/R	-16	24	9	4.07	≤ .001	4
Cingulate/caudate	L/R	-9/21	30	-6/0	5.07	≤ .001	7
Cingulate gyrus	R	18	-12	48	5.10	≤ .001	7
Anterior cingulate	R	3	3	24	4.86	≤ .001	6
Inferior colliculus	L	-3	-33	-15	4.18	≤ .001	6
Thalamus	R	9	-9	24	6.17	≤ .001	7
Amygdala	L	-6	0	-18	3.95	≤ .001	6
Hippocampus	L	-30	-18	-18	5.06	≤ .001	4
Anterior insula	R	27	33	-3	3.80	0.002	4
Medial frontal gyrus	L	-12	45	24	6.24	≤ .001	7
Orbitofrontal gyrus	R	24	42	-3	3.91	≤ .001	7
IPS	R	21	-36	30	6.10	≤ .001	7
SII	R	51	-39	18	4.61	≤ .001	7

HFN= Highly familiar neutral.
